# Two New Pyranoxanthones from *Mesua beccariana* (Guttiferae)

**DOI:** 10.3390/molecules15106733

**Published:** 2010-09-27

**Authors:** Soek Sin Teh, Gwendoline Cheng Lian Ee, Mawardi Rahmani, Wei Chung Sim, Siau Hui Mah, Siow Hwa Teo

**Affiliations:** Department of Chemistry, Faculty of Science, Universiti Putra Malaysia, 43400 Serdang, Selangor, Malaysia

**Keywords:** mesuarianone, mesuasinone, pyranoxanthone, *Mesua beccariana*, Guttiferae

## Abstract

Our recent study on the stem bark extracts of *Mesua beccariana* has led to the isolation of two new pyranoxanthones, mesuarianone (**1**) and mesuasinone (**2**), two anthraquinones, 4-methoxy-1,3,5-trihydroxyanthraquinone (**3**) and 2,5-dihydroxy-1,3,4-trimethoxyanthraquinone (**4**), one coumarin, mammea *A/AB* (**5**) and three common triterpenes, stigmasterol (**6**), friedelin (**7**) and betulinic acid (**8**). Structural elucidations of these compounds were achieved using 1D and 2D NMR and MS techniques. This is the first report on the phytochemistry of *Mesua beccariana*.

## 1. Introduction

**Figure 1:**
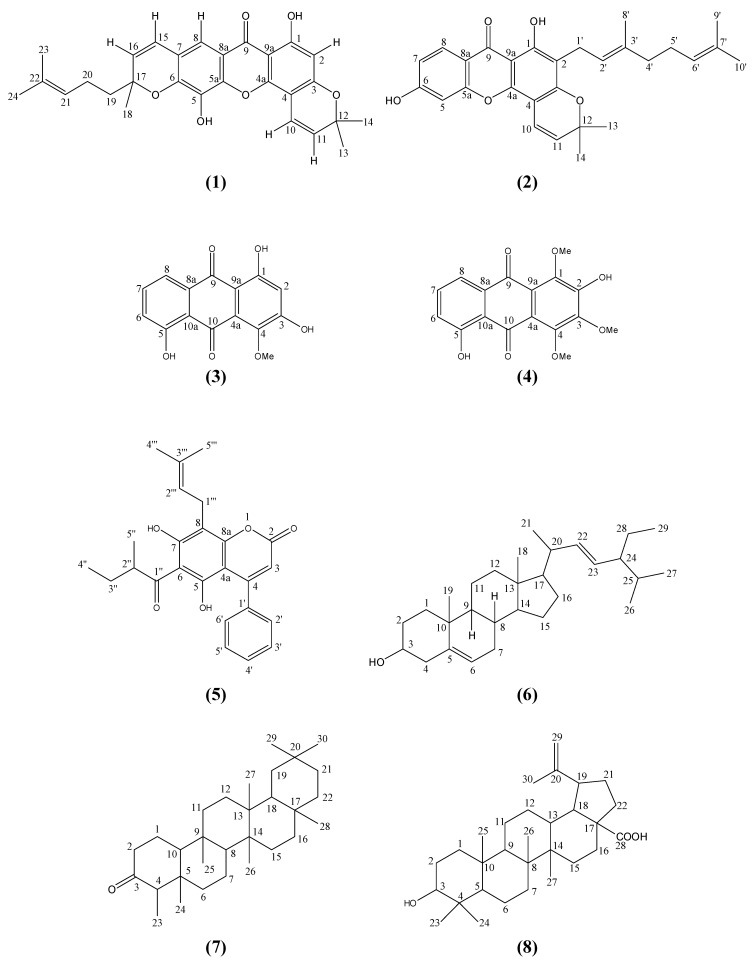
Structures of compounds.

*Mesua* is a flowering genus consisting of about 48 species indigenous to south tropical Asia [1]. It is commonly known to the locals in Malaysia as ironwood due to its very hard and heavy wood, which is used for railroad ties and structural timber. Although there are some phytochemical reports on the genus *Mesua*, focused on *Mesua ferrea* [2,3,4,5], and a few on *Mesua racemosa* [6], *Mesua thwaitesii* [7] and *Mesua daphnifolia* [8,9], no study has been carried out on the chemistry of *Mesua beccariana*. These reports revealed the presence of xanthones, coumarins, biflavanoids and triterpenoids. This paper reports our recent phytochemical discovery of two new pyranoxanthones – mesuarianone (**1**) and mesuasinone (**2**) from the stem bark of *Mesua beccariana* (Figure 1). 

## 2. Results and Discussion

Compound **1**, mesuarianone (Figure 1), which was isolated from the hexane stem bark extract of *Mesua beccariana*, is a yellow powder with melting point 166-167 °C. The EIMS spectrum gave a molecular ion (M^+^) peak at *m/z* 460, while the HRESIMS spectrum gave 460.1827 (calc’d. 460.1886) consistent with the molecular formula C_28_H_28_O_6_. The IR showed ν_max_ : 3392 (OH), 2971 (CH_3_), 2923 (CH_2_), 1639 (C=O), 1574 and 1475 (C=C aromatic) cm^-1^. The UV maximum absorptions at 208 (6.41), 280 (6.49), 333.8 (6.28) nm were characteristic of a xanthone skeleton. 

The ^1^H- and ^13^C-NMR spectral information of **1** revealed the existence of 28 hydrogens and 28 carbons, supporting the molecular formula above. These spectroscopic data, along with the DEPT analysis indicated the presence of a xanthone skeleton with all positions substituted, except for C-2 (δ 99.6) and C-8 (δ 113.7). Further examination of the DEPT experiment revealed the existence of four non-oxygenated aromatic carbons which are C-4 (δ 101.6), C-7 (δ 117.8), C-8a (δ 114.8) and C-9a (δ 103.4), along with six oxygenated aromatic carbons which are C-1 (δ 163.3), C-3 (δ 160.7), C-4a (δ 151.8), C-5a (δ 145.3), C-5 (δ 132.5) and C-6 (δ 145.3). The presence of a chelated carbonyl group was indicated by a downfield carbon signal at δ 180.5 (C-9). In the ^1^H-NMR spectrum, a chelated phenolic hydroxyl group (δ 13.05, 1H, s) and two one-proton aromatic singlets (δ 6.23 and 7.44) were observed.

The HMBC spectrum demonstrated the ^2^*J* and ^3^*J* multiple bond ^1^H-^13^C correlations in the molecule. The sharp singlet in the downfield region at δ 13.05 indicated a chelated hydroxyl group. Its multiple-bond correlations to δ 99.6 (C-2), 103.4 (C-9a) and 163.3 (C-1) in the HMBC spectrum confirmed its position at C-1. Moreover, ^3^*J* correlations between δ 7.44 (H-8) and C-9 (δ 180.5) and C-5a (δ 145.3) were clearly seen. Meanwhile, the singlet at δ 6.23 (H-2) gave a ^3^*J* correlation to δ 103.4 (C-9a) and a ^2^*J* correlation to δ 160.7 (C-3). This confirmed the position of these two aromatic methines to be at C-8 and C-2, respectivel. 

**Figure 2:**
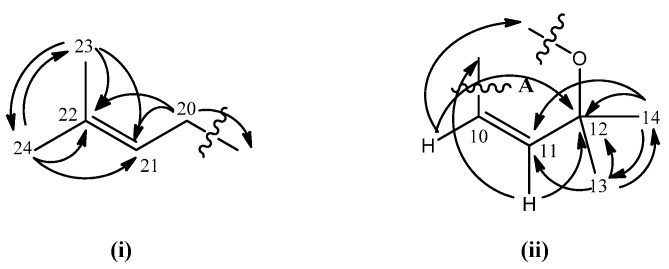
Prenyl moiety (i) and pyrano ring system (ii) and their HMBC correlations.

**Figure 3:**
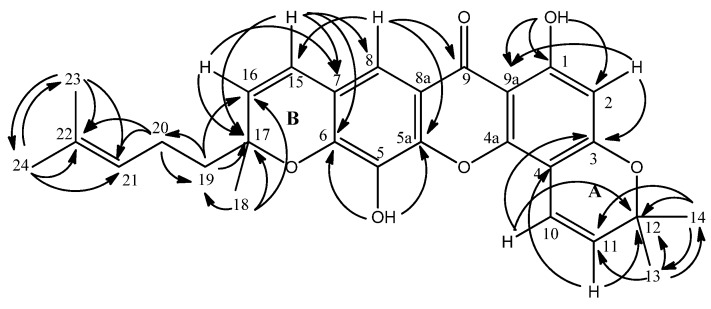
^2^*J* and ^3^*J* correlations between ^1^H and ^13^C in HMBC analysis.

The characterization of chemical shifts for protons at δ 2.10 (*m*, 2H, H-20), 5.06 (t, 1H, H-21), 1.53 (s, 3H, H-23), 1.63 (s, 3H, H-24) and carbons at δ 22.9 (C-20), 123.6 (C-21), 132.6 (C-22), 17.9 (C-23) and 25.9 (C-24) suggested the presence of a prenyl moiety in the molecule (Figure 2). This hypotesis was further confirmed by the establishment of the connectivity of the two vinylic methyl proton singlets at δ 1.53 (H-23) and δ 1.63 (H-24) to their neighbouring carbons at δ 132.3 (C-22) and 123.6 (C-21) in the HMBC spectrum. On the other hand, HMBC experiment indicated the linkage between two aliphatic methyls resonating at δ 1.46 (H-13 & H-14) to carbon signals at δ 78.4 (C-12) and 127.3 (C-11). Long-range ^3^*J* and ^2^*J* correlations between doublet protons at δ 6.87 (H-10, *J* = 10.1 Hz) and 5.56 (*J* = 10.1 Hz, H-11) and the carbon signal at δ 78.4 (C-12) respectively were also seen. These data together with the COSY spectrum analysis suggested the existence of a pyrano ring (ring **A**; Figure 2). Meanwhile, HMBC correlations of H-19 (δ 1.79) and H-18 (δ 1.48) to C-17 (δ 81.7) and the two ortho coupled vinylic protons H-15 and H-16 (δ 6.46 and 5.65) to C-17 (δ 81.7) were also observed (Figure 3). The latter correlation pattern is similar to that of ring **A**, thus implying another pyrano ring (ring **B**) in the molecule. However, the latter pyrano ring carries only one methyl group. Two methylene hydrogens which gave a multiplet signal at δ 2.10 (H-20) demonstrated a ^2^*J* correlation to the carbon signal at δ 41.8 (C-19). This information allowed the placement of the prenyl moiety at C-19 which is attached to the pyrano ring **B**. The HMBC spectrum confirms the attachment of C-19 to C-17. ^2^*J* and ^3^*J* linkages of H-19 to C-17 and C-16 respectively were observed.

The connectivity of δ 6.87 (H-10) to C-3 (δ 160.7) and δ 5.56 (H-11) to C-4 (δ 101.6) as observed in the HMBC experiment led to the attachment of pyrano ring A to position C-3 and C-4 in the xanthone skeleton. Similarly, the other prenylated pyrano ring (ring **B**) is attached to position C-6 and C-7 following the observation of ^3^*J* and ^2^*J* long-range connectivity between H-15 (δ 6.46) and C-6 (δ 145.3), C-7 (*δ* 117.8) and C-8 (*δ* 113.7). The non-chelated hydroxyl singlet at δ 5.52 gave ^3^*J* linkages to two quartenary aromatic carbons with overlapped chemical shifts at δ 145.3 (C-5a and C-6). These evidences led to the assignment of the hydroxyl group at C-5 (δ 132.5) (Figure 3).

Hence, the structure of compound **1** was elucidated to be 1,5-dihydroxy-6’,6’-dimethylpyrano[2’,3’: 3,4]-6”-(2-methyl-2-pentenyl)-6”-methylpyrano[2”,3”:6,7]-xanthone and named as mesuarianone. The NMR data of this compound are summarized in Table 1.

Compound **2**, mesuasinone (Figure 1), which was obtained as a yellow solid with m.p. 118-119 °C, and molecular formula C_28_H_30_O_5_ (EIMS *m/z* 446), as confirmed by HREISIMS 446.2041 (calc’d. 446.2093) had UV and IR spectral data suggestive of a xanthone derivative. The UV spectrum gave maximum absorptions at 208 (3.82), 254 (3.96), 274.0 (3.93), 334 (3.53) nm, while the IR spectrum showed absorptions at 3229 (OH), 2925 (CH_3_), 2865 (CH_2_), 1640 (C=O), 1576 and 1496 (C=C aromatic) cm^-1^.

The ^13^C-NMR signals showed the presence of a carbonyl carbon (δ 180.7) along with the presence of twelve quaternary carbons (δ 80.7, 100.3, 103.0, 111.8, 121.2, 131.7, 132.1, 144.1, 144.2, 149.2, 158.9, 160.6), seven methines (δ 115.4, 117.1, 120.1, 121.9, 123.6, 123.9, 126.1), three methylenes (δ 21.2, 22.8, 41.8) and five methyls (δ 17.6, 17.9, 25.6, 25.8, 27.1). The ^13^C and DEPT NMR spectra of compound **2** showed some similarity to that of **1**, suggesting **2** also has a pyranoxanthone structure. 

Moreover, the ^1^H-NMR spectra also supported the existence of a pyrano ring. Two vinylic protons were observed at δ 6.83 (1H, d, *J* = 10.1 Hz, H-10) and 5.58 (1H, d, *J* = 10.1 Hz, H-11) along with two methyl signals at δ 1.45 (3H, s, H-14) and 1.66 (3H, s, H-13). The methyl protons H-13 (δ 1.66) demonstrated ^3^*J* correlations with δ 27.1 (C-14) and H-14 (δ 1.45) showed ^2^*J* correlations with δ 80.7 (C-12) and ^3^*J* correlations with δ 126.1 (C-11) in the HMBC experiment. This pyrano ring was connected to the xanthone skeleton at C-3 and C-4 as evidenced by the ^3^*J* HMBC correlations between the vinylic proton at δ 6.83 (H-10) and δ 149.2 (C-4a) and δ 158.9 (C-3), while the other vinylic proton at δ 5.58 (H-11) correlates to δ 100.3 (C-4) via another ^3^*J* HMBC correlation.

The ^1^H NMR spectra also gave a sharp singlet at δ 13.02 indicating a chelated hydroxyl group, while the broad singlet at δ 5.73 was due to the free hydroxyl group. In the HMBC spectra, the chelated hydroxyl proton (δ 13.02) showed cross-peaks with three quaternary carbon signals at δ 103.0 (C-9a, ^3^*J*), 111.8 (C-2, ^3^*J*) and 160.6 (C-1, ^2^*J*). The deshielded carbon signal at 160.6 (C-1) was found to be attached to the hydroxyl group.

**Figure 4:**
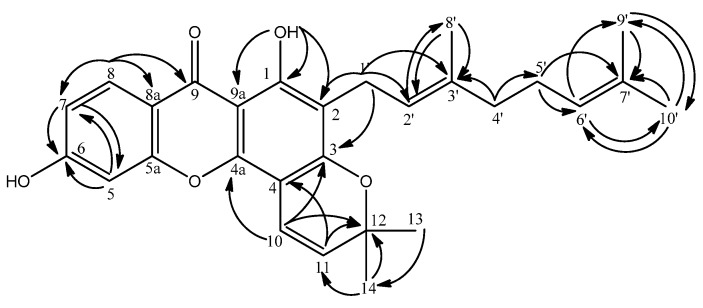
HMBC Correlations in Compound **2**.

Three aromatic protons which are arranged in the ortho and meta positions were revealed by the resonances at δ 7.22 (1H, d, *J* = 8.3 Hz), 7.77 (1H, d, *J* = 8.3 Hz) and 7.29 (1H, s). The lowest-field aromatic proton (δ 7.77) was assigned to H-8 due to the anisotropic effect of the carbonyl group. This was supported by the ^3^*J* and ^2^*J* HMBC correlations of H-8 to a carbonyl signal at δ 180.7 (C-9) and two aromatic carbons C-8a (δ 121.2) and C-7 (δ 120.1), while its ortho-coupled proton H-7 correlates to δ 123.9 (C-5) and 121.2 (C-8a) as shown in Figure 4. The position of the proton resonating at δ 7.29 (1H, *s*) was confirmed to be at C-5 via its HMBC correlations to the carbon signals at δ 120.1 (C-7, ^3^*J*) and 144.1 (C-6, ^2^*J*).

**Figure 5:**
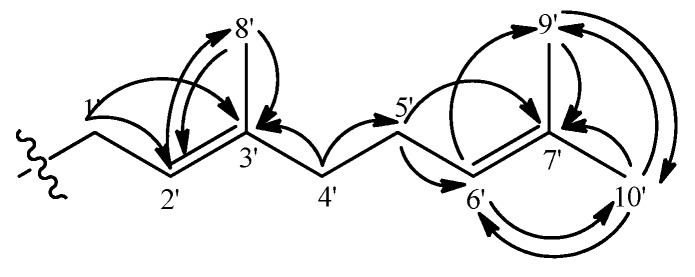
The geranyl moiety and its long-range Heteroatom Correlations.

The ^1^H-NMR spectra revealed a geranyl moiety at δ 3.36 (2H, d, *J* = 7.3 Hz, H-1’), 5.24 (1H, t, *J* = 7.3 Hz, H-2’), 1.84 (2H, m, H-4’), 2.10 (2H, m, H-5’), 5.09 (1H, t, *J* = 6.4 Hz, H-6’), 1.80 (3H, s, H-9’), 1.68 (3H, s, H-10’), 1.57 (3H, s, H-8’). This was further confirmed using the long-range correlation system observed in the HMBC spectra as illustrated in Figure 5. The benzylic allylic methylene protons (δ 3.36, H-1’) of the geranyl group showed correlations with δ 111.8 (C-2, ^2^*J*) and 158.9 (C-3, ^3^*J*) allowing its placement at C-2.

Hence, the structure of compound **2** was elucidated to be 1,6-dihydroxy-2-(3’,7’-dimethyl-2’,6’-octenyl)-6”,6”-dimethylpyrano-[2”,3”:3,4]-xanthone and named mesuasinone. The NMR data of this compound are summarized in Table 1.

4-Methoxy-1,3,5-trihydroxyanthraquinone (3) [10], 2,5-dihydroxy-1,3,4-trimethoxyanthraquinone (4) [10], mammea A/AB (5) [11], stigmasterol (6) [13], friedelin (7) [13] and betulinic acid (8) [16,17] were identified by comparing their spectral data with literature.

## 3. Experimental 

### 3.1. General

Infrared spectra were measured using the universal attenuated total reflection (UATR) technique on a Perkin-Elmer 100 Series FT-IR spectrometer. EIMS were recorded on a Shimadzu GCMS-QP5050A spectrometer. NMR spectra were obtained using a Unity JEOL 400 MHz FTNMR spectrometer using CDCl_3_ as solvent and tetramethylsilane (TMS) as internal standard. Ultraviolet spectra were recorded in EtOH on a Shimadzu UV-160A, UV-Visible Recording Spectrophotometer.

### 3.2. Plant Material

The stem bark of *Mesua beccariana* was collected from the Sri Aman district in Sarawak, Malaysia. The plant material was identified by Associate Professor Dr Rusea Go, Biology Department, Faculty of Science, Universiti Putra Malaysia.

### 3.3. Extraction and Isolation

The air-dried and powdered sample was extracted successively with *n*-hexane, dichloromethane, ethyl acetate and methanol. The extracts were dried under reduced pressure using a rotary evaporator to yield hexane (15.6 g), dichloromethane (21.2 g), ethyl acetate (15.8 g) and methanol (80.5 g) extracts. Each of these extracts was chromatographed over a silica gel column using a stepwise gradient system (hexane/chloroform, chloroform/ethyl acetate, and ethyl acetate/methanol). The column chromatography of the hexane extract gave mesuarianone (**1**, 110 mg), mesuasinone (**2**, 76 mg) and friedelin (**7**, 20 mg). Meanwhile, the dichloromethane extract gave stigmasterol (**6**, 23 mg) and betulinic acid (**8**, 45 mg) while the ethyl acetate extract gave two anthraquinones and a coumarin identifed as 4-methoxy-1,3,5-trihydroxyanthraquinone (**3**, 9 mg), 2,5-dihydroxy-1,3,4-trimethoxy-anthraquinone (**4**, 8 mg) and mammea *A/AB* (**5**, 11 mg). 

### 3.4. Spectral Data

*Mesuarianone* (**1**). Yellow solid. m.p. 166-167 °C. ${\left[\alpha \right]}_{589}^{25}$(CHCl_3_) +22.5. UV $\lambda_{\max}^{\mathrm{EtOH}}$ nm (log ε): 208 (6.41), 280 (6.49), 333.8 (6.28). IR ν_max_ (cm^-1^): 3392, 2971, 2923, 1639, 1574, 1475. For ^1^H- and ^13^C- NMR spectra, see Table 1. MS *m/z* (rel. int.): 460 [M^+^] (39), 445 (100), 377 (89), 361 (10), 347 (11), 323 (12), 203 (8), 181 (61), 91 (6), 77 (5), 69 (27), 55 (9). 

*Mesuasinone* (**2**). Yellow solid. m.p. 118-119 °C. UV $\lambda_{\max}^{\mathrm{EtOH}}$ nm (log ε): 208 (3.82), 254 (3.96), 274.0 (3.93), 334 (3.53). IR ν_max_ (cm^-1^): 3229, 2925, 2865, 1640, 1576, 1496. For ^1^H- and ^13^C-NMR spectra, see Table 1. MS *m/z* (rel. int.): 446 [M^+^] (14), 431 (4), 391 (8), 363 (100), 307 (21), 154 (12), 69 (10). 

*4-Methoxy-1,3,5-trihydroxyanthraquinone* (**3**). Orange solid. UV $\lambda_{\max}^{\mathrm{MeOH}}$ nm: 279, 320, 425, 470, 485. IR ν_max_ (cm^-1^): 3420, 2920, 2860, 1720, 1630, 1470. MS *m/z* (rel. int.): 286 [M^+^] (100), 268 (87), 257 (10), 243 (38), 212 (27), 180 (30). The ^1^H- and ^13^C-NMR spectral data are consistent with published data [10].

*2,5-Dihydroxy-1,3,4-trimethoxyanthraquinone* (**4**). Orange solid. UV $\lambda_{\max}^{\mathrm{MeOH}}$ nm: 218,276,410. IR ν_max_ (cm^-1^): 3400, 2920, 2840, 1660, 1630,1540. MS *m/z* (rel. int.): 330 [M^+^] (100), 315 (60), 312 (5), 297 (20), 287 (22), 272 (24), 227 (20), 58 (23). The ^1^H- and ^13^C-NMR spectral data are consistent with published data [10]. 

*Mammea A/AB* (**5**). Colourless solid. UV $\lambda_{\max}^{\mathrm{EtOH}}$ nm: 283, 337. MS *m/z* (rel. int.): 406 [M^+^] (2), 392 (18), 377 (10), 349 (100), 293 (10). The ^1^H- and ^13^C-NMR (CDCl_3_) spectral data are consistent with the published data [11]. 

*Stigmasterol* (**6**). White needles. m.p. 155-156 °C (Lit. 168-169 °C) [12]. IR ν_max_ (cm^-1^): 3399, 2939, 1457, 1374. MS *m/z* (rel. int.): 412 [M^+^] (60), 394 (7), 369 (7), 351 (15), 300 (26), 271 (38), 255 (40), 159 (41), 145 (42), 133 (45), 123 (31), 105 (42), 95 (44), 83 (77), 69 (62), 55 (100). The ^1^H- and ^13^C- NMR (CDCl_3_) spectral data are consistent with literature [13]. 

*Friedelin* (**7**). White needles. m.p. 245-246 °C (Lit. 260-263 °C) [14]. IR ν_max_ (cm^-1^): 1714, 1457, 1380. MS *m/z* (rel. int.): 426 [M^+^] (12), 411 (6), 302 (11), 273 (16), 246 (17), 231 (16), 218 (19), 205 (20), 191 (18), 179 (19), 163 (27), 149 (18), 137 (24), 123 (60), 109 (64), 95 (76), 81 (63), 69 (100), 55 (75), 41 (44). The ^1^H- and ^13^C-NMR spectral data are consistent with literature [13]. 

*Betulinic acid* (**8**). White solid. m.p. 290-291 °C (Lit. 291-292 °C) [15]. IR ν_max_ (cm^-1^): 3460, 2939, 1687, 1453, 1375. MS *m/z* (rel. int.): 456 [M^+^] (7), 438 (4), 248 (40), 207 (48), 189 (100), 69 (17). The ^1^H- and ^13^C-NMR spectral data are consistent with published data [16,17]. 

## 4. Conclusions

Two new pyranoxanthones, mesuarianone (**1**) and mesuasinone (**2**), along with two anthraquinones, a coumarin, and three triterpenes were isolated from the stem bark of *Mesua beccariana*. Future work on the biological activities such as anti-cancer and anti-oxidant properties will be carried out in due course.

## Figures and Tables

**Table 1:** ^1^H-NMR (400 MHz, CDCl_3_) and ^13^C-NMR (100 MHz, CDCl_3_) data of mesuarianone (**1**) and mesuasinone (**2**).

Position	**1**		**2**	
	^1^H(*δ*)	^13^C(*δ*)	^1^H(*δ*)	^13^C(*δ*)
1	-	163.3	-	160.6
2	6.23 (*s*)	99.6	-	111.8
3	-	160.7	-	158.9
4	-	101.6	-	100.3
4a	-	151.8	-	149.2
5	-	132.5	7.29 (*s*)	123.9
5a	-	145.3	-	144.2
6	-	145.3	5.73 (*s*)	144.1
7	-	117.8	7.22 (*d*, 8.3)	120.1
8	7.44 (*s*)	113.7	7.77 (*d*, 8.3)	117.1
8a	-	114.8	-	121.2
9	-	180.5	-	180.7
9a	-	103.4	-	103.0
10	6.87 (*d*, 10.1)	115.4	6.83 (*d*, 10.1)	115.4
11	5.56 (*d*, 10.1)	127.3	5.58 (*d*, 10.1)	126.1
12	-	78.4	-	80.7
13	1.46 (*s*, 3H)	28.5	1.66(*s*, 3H) 1.45(*s*)	25.6 27.1
14	1.46 (*s*, 3H)	28.5	1.66(*s*, 3H)	
15	6.46 (*d*, 10.1)	122.2		
16	5.65 (*d*, 10.1)	130.0		
17	-	81.7		
18	1.48 (*s*, 3H)	27.5		
19	1.79 (*m*, 2H)	41.8		
20	2.10 (*m*, 2H)	22.9		
21	5.06 (*t*, 7.3)	123.6		
22		132.3		
23	1.53 (*s*, 3H)	17.9		
24	1.63 (*s*, 3H)	25.9		
1’			3.36 (*d*, 7.3, 2H)	21.2
2’			5.24 (*t*, 7.3)	121.9
3’			-	132.1
4’			1.84 (*m*, 2H)	41.8
5’			2.10 (*m*, 2H)	22.8
6’			5.09 (*t*, 6.4)	123.6
7’			-	131.7
8’			1.57 (*s*, 3H)	17.6
9’			1.80 (*s*, 3H)	17.9
10’			1.68 (*s*, 3H)	25.8
1-OH	13.05 (*s*)	-	13.02 (*s*)	-
5-OH	5.52 (*s*)	-		
6-OH			5.73 (*s*)	-

*Coupling constants (*J*) in Hz are stated in parentheses.

## References

[1] Ee G.C.L., Lim C.K., Rahmat A., Lee H.L. (2005). Cytotoxic activities of chemical constituents from *Mesua daphnifolia*. Trop. Biomed..

[2] Walia S., Mukerjee S.K. (1984). Ferrxanthone, a 1,3,5,6-tetraoxygenated xanthone from *Mesua ferrea*. Phytochemistry.

[3] Raju M.S., Srimannarayan G., Rao N.V.S. (1976). Structure of mesuaferrone-B a new biflavonone from the stamens of *Mesua ferrea Linn*. Tetrahedron Lett..

[4] Chow Y.L., Quon H.H. (1968). Chemical constituents of the heartwood of *Mesua ferrea*. Phytochemistry.

[5] Govindachari T.R., Pai B.R., Subramaniam P.S., Rao U.R., Muthukumaraswa N. (1967). Constituents of *Mesua ferrea* L.-mesuaxanthone A and mesuaxanthone B. Tetrahedron.

[6] Morel C., Guilet D., Oger J.M., Seraphin D., Sevenet T., Wiart C., Hadi A.H.A., Richomme P., Bruneton J. (1999). 6-Acylcoumarins from *Mesua racemosa*. Phytochemistry.

[7] Bandaranayake W.M., Selliah S.S., Sultanbawa M.U.S., Games D.E. (1975). Xanthones and 4-phenylcoumarins of *Mesua thwaitesii*. Phytochemistry.

[8] Ee G.C.L., Lim C.K., Cheow Y.L., Sukari M.A. (2005). Xanthones and triterpenoids from *Mesua daphnifolia* and *Garcinia maingayi*. Malaysian J. Sci..

[9] Ee G.C.L., Lim C.K., Ong G.P., Sukari M.A., Lee H.L. (2006). Daphnifolin, a new xanthone from *Mesua daphnifolia*. J. Asian Nat. Prod. Res..

[10] Wijnsma R., Verpoorte R., Mulder-Krieger T., Svendsen A.B. (1984). Anthraquinones in callus cultures of Cinchona ledgeriana. Phytochemistry.

[11] Verotta L., Lovaglio E., Vidari G., Finzi P.V., Neri M.G., Raimondi A., Parapini S., Taramelli D., Riva A., Bombardelli E. (2004). 4-Alkyl- and 4-phenylcoumarins from *Mesua ferrea* as promising multidrug resistant antibacterials. Phytochemistry.

[12] Jamaluddin F., Mohamed S., Lajis M.N. (1994). Hypoglycaemic effect of Parkia speciosa seeds due to the synergistic action of [beta]-sitosterol and stigmasterol. Food Chem..

[13] Ee G.C.L., Kua A.S.M., Lim C.K., Jong V., Lee H.L. (2006). Inophyllin A, a new pyranoxanthone from *Calophyllum inophyllum* (Guttiferae). Nat. Prod. Res.: Formerly Nat. Prod. Lett..

[14] Klass J., Tinto W.F., McLean S., Reynolds W.F. (1992). Friedeland Triterpenoids from *Peritassa compta*: Complete 1H and 13C Assignments by 2D nmr Spectroscopy. J. Nat. Prod..

[15] Kim D.S.H.L., Chen Z., Nguyen V.T., Pezzuto J.M., Qiu S., Lu Z.-Z. (1997). A Concise Semi-Synthetic Approach to Betulinic Acid from Betulin. Synth. Commun..

[16] Tapondjou A.L., Miyamoto T., Lacaille-Dubois M.-A. (2006). Glucuronide triterpene saponins from Bersama engleriana. Phytochemistry.

[17] Galgon T., Höke D., Dräger B. (1999). Identification and quantification of betulinic acid. Phytochem. Anal..

